# COVID-19 Vaccine-Induced Myocarditis and Pericarditis: Towards Identification of Risk Factors

**DOI:** 10.5334/gh.1252

**Published:** 2023-07-31

**Authors:** Laura C. Zwiers, David S. Y. Ong, Diederick E. Grobbee

**Affiliations:** 1 Julius Clinical, Zeist, The Netherlands; 2 Julius Global Health, Julius Center for Health Sciences and Primary Care, University Medical Center Utrecht, Utrecht University, Utrecht, The Netherlands; 3 Department of Medical Microbiology and Infection Control, Franciscus Gasthuis & Vlietland, Rotterdam, The Netherlands

**Keywords:** COVID-19, Vaccination, Myocarditis, Pericarditis

**Publisher’s Note:** A correction article relating to this paper has been published and can be found at https://globalheartjournal.com/articles/10.5334/gh.1276/.

**Graphical Abstract**

**Figure:**
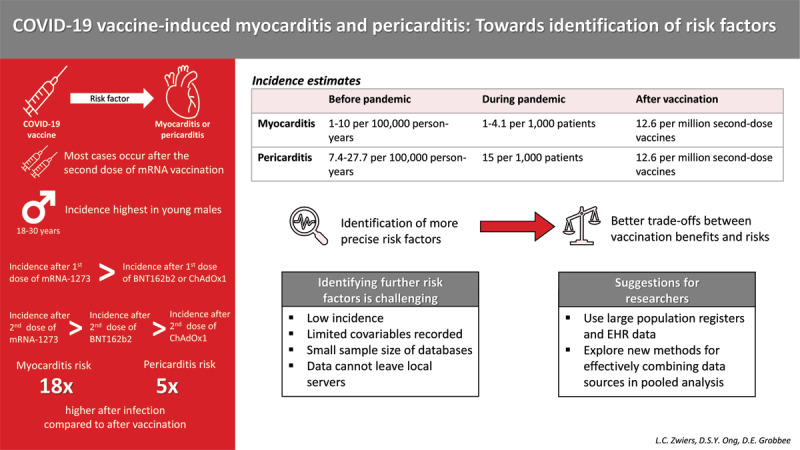

The large impact of the coronavirus disease, 2019 pandemic (COVID-19) led to an unprecedented speed of vaccine development, with vaccines approved for administration within a year after emergence of the severe acute respiratory syndrome, coronavirus 2 (SARS-CoV-2). Since the administration of the first dose, over 12 billion vaccine doses have been administered worldwide [1]. With rolling out vaccination programmes on such a large scale also came reports of adverse events. Myocarditis and pericarditis were among the reported complications, which were especially related to the two available mRNA COVID-19 vaccines [2,3,4], but have also been reported following the ChAdOx1 adenovirus vaccine [5].

These vaccines are not the first medical products for which myocarditis and pericarditis have been reported as adverse events. For instance, smallpox and influenza vaccinations are known potential causes, for which myocarditis and pericarditis occurred most often in young males, and in individuals with a smoking history, physical limitations, or lower self-reported health [6]. Another known potential cause is the use of clozapine, for which demographic characteristics and factors in individuals’ medical history were found unrelated to the occurrence of myocarditis and pericarditis [7].

Prior to the pandemic, the global incidence of myocarditis was 1 to 10 cases per 100,000 person-years [8]. Incidence rates increased during the pandemic due to COVID-19-induced myocarditis, with an incidence of 1 to 4.1 cases per 1,000 hospitalized patients [8,9,10,11], although these estimates might suffer from under-reporting [12]. For pericarditis, incidence estimates ranged between 7.4 and 27.7 per 100,000 person-years prior to the pandemic [13,14,15]. A cohort study of patients with COVID-19 reported that 1.5% of patients develop new-onset pericarditis [16], but additional incidence estimates for pericarditis during the pandemic are lacking. Certain types of COVID-19 vaccinations were found to be associated with an increased risk of developing myocarditis and pericarditis, although this risk was lower in comparison to after SARS-CoV-2 infection. Patone et al. [5] estimated that there is an increased risk of myocarditis following the first dose of ChAdOx1, BNT162b2, and mRNA-1273 with incidence rate ratios (IRRs) of 1.29, 1.31 and 2.97, respectively. An increased risk after the second dose was found for both mRNA vaccines but not for ChAdOx1. For the BNT162b2 vaccine the IRR was estimated to be 1.30 and for mRNA-1273 the IRR was 9.84. For mRNA vaccines only, combined incidence of myocarditis and pericarditis was estimated to be 12.6 cases per one million second doses among individuals aged 12 to 39 [17]. For myocarditis alone, the Vaccine Adverse Reporting System (VAERS) indicated that, in 2021, approximately 4.8 cases per million doses of mRNA vaccines administered occurred among individuals aged 12 or older [18].

Previous studies have shown that incidence of post-vaccination myocarditis and pericarditis is highest in adolescent and young adult males and that symptoms most often occur approximately three days after the second dose of the mRNA COVID-19 vaccine [19,20,21,22], and after the first dose of the ChAdOx1 vaccine [5]. These conclusions are mainly based on case reports and case series reported to, amongst others, the VAERS, the UK Yellow Card scheme and EudraVigilance (see e.g. [19]). Higher incidences of myocarditis among young males were also already reported prior to the COVID-19 pandemic [23], indicating that these individuals may be at higher risk of myocarditis in general. Between the different vaccines, most studies conclude that the risk of developing myocarditis is highest after receiving mRNA-1273 vaccination [5,19,21,24]. Moreover, mRNA vaccinations in general were associated with a higher risk of myocarditis compared to adenovirus vaccines [25]. However, some studies have argued that the risk after receiving BNT162b2 vaccination is higher than after mRNA-1273 vaccination [22], that the risk is similar for BNT162b2 and ChAdOx1 after the first dose [5], or that the risk for mRNA-173 was only higher than that for BNT162b2 in specific age groups [20]. For example, among males aged 18 to 39, 19.2 cases per million doses of mRNA-1273 were reported in comparison to 16.5 per million doses of BNT162b2. For females of the same age, the reported rates were 3.1 and 1.4, respectively [20]. Some estimated incidences, based on existing cohort studies, are reported in Table 1.

**Table 1:** Estimated incidences of myocarditis and pericarditis after COVID-19 vaccination in existing cohort studies.


OUTCOME	ESTIMATED INCIDENCE	POPULATION	SOURCE

*Vaccine: BNT162b2*			

Myocarditis and pericarditis	1.71 per 100,000 person-days^1^	Males aged 18–25	Wong et al. [21]

Myocarditis and pericarditis	0.52 per 100,000 person-days^1^	Females aged 18–25	Wong et al. [21]

Myocarditis and myopericarditis	1.5 per 100,000 individuals^2^	Males (all ages)	Husby et al. [24]

Myocarditis and myopericarditis	1.3 per 100,000 individuals^2^	Females (all ages)	Husby et al. [24]

Myocarditis and myopericarditis	1.6 per 100,000 individuals^2^	Individuals aged 12–39	Husby et al. [24]

Myocarditis and myopericarditis	1.0 per 100,000 individuals^2^	Individuals aged 12–17	Husby et al. [24]

Myocarditis	IRR after first dose: 1.31^3^	All individuals	Patone et al. [5]

	IRR after second dose: 1.30^3^		

*Vaccine: mRNA-1273*			

Myocarditis and pericarditis	2.17 per 100,000 person-days^1^	Males aged 18–25	Wong et al. [21]

Myocarditis and pericarditis	0.45 per 100,000 person-days^1^	Females aged 18–25	Wong et al. [21]

Myocarditis and myopericarditis	6.3 per 100,000 individuals^2^	Males (all ages)	Husby et al. [24]

Myocarditis and myopericarditis	2.0 per 100,000 individuals^2^	Females (all ages)	Husby et al. [24]

Myocarditis and myopericarditis	5.7 per 100,000 individuals^2^	Individuals aged 12–39	Husby et al. [24]

Myocarditis	IRR after first dose: 2.97^3^	All individuals	Patone et al. [5]

	IRR after second dose: 9.84^3^		

*Vaccine: ChAdOx1*			

Myocarditis and pericarditis	0.18 per 100,000 individuals^4^	All individuals	Rahman et al. [25]

Myocarditis	IRR after first dose: 1.29^3^	All individuals	Patone et al. [5]

	IRR after second dose: 1.00^3^		


^1^ Estimate only for incidence after second dose of the vaccine; ^2^ Incidence per 100,000 vaccinated individuals within 28 days of vaccination; ^3^ Incidence Rate Ratios (IRRs) for 1–28 days following vaccination, included here as incidence estimates were not available; ^4^ Incidence per 100,000 vaccinated individuals within 21 days of vaccination.

While it has been well-established that the risk of post-vaccination myocarditis and pericarditis is highest in young males, possible risk factors other than age and sex are under-investigated. One reason for this is the low number of observed events, due to the low incidence rate. This makes it difficult to simultaneously investigate a large number of variables, as results can be erroneous when too few events are observed relative to the number of investigated variables [26]. When additional variables in multivariable models for the study of post-vaccination myocarditis and pericarditis were included, these were only considered as possible confounders that needed to be controlled for rather than investigated as potential risk factors. Studies by Karlstadt et al. [27] and Husby et al. [24] adjusted for age and sex, as well as comorbidities such as pulmonary disease, cardiovascular disease, diabetes, and cancer. However, it was not reported whether these comorbidities were associated with the risk of myocarditis and pericarditis.

Because of the low incidence of post-vaccination myocarditis and pericarditis, as well as the high recovery rates, most experts in the field argue that the risks of these adverse reactions do not outweigh the benefits of vaccination, and that vaccination should therefore be encouraged [8]. The risk of myocarditis and pericarditis is also substantially higher after infection than after vaccination (i.e. 18 times higher for myocarditis, and five times higher for pericarditis, respectively) [11]. However, recent research by Kracalik and colleagues suggested that in the longer term, the psychosocial burden of post-vaccination myocarditis should not be ignored [28]. Acquired immunity through vaccinations and previous infections, as well as virus mutations that are associated with decreased virulence and impact, have decreased the importance of widespread restrictions to curb the spread of COVID-19. This has fuelled the debate whether the benefits of repeat vaccinations still outweigh the risks of adverse events for all individuals. However, current knowledge on risk factors for the development of myocarditis is too limited to make proper trade-offs between risks and benefits. This is especially because it seems unlikely that the risks outweigh vaccination benefits for everyone in the currently identified at-risk groups, namely all young males.

To more accurately identify which individuals are at highest risk of developing post-vaccination myocarditis and pericarditis, several issues regarding methodological study design need to be overcome. First, large datasets containing information about individuals’ vaccination history and the outcomes of interest, but also about other potentially relevant covariables, such as medical history and lifestyle, are needed. Frequently used databases for research on vaccine-related adverse events, such as the VAERS database, contain reports of suspected adverse events, but do not have a control group. Moreover, only a very limited number of covariables is available in these databases [29]. Studies using electronic health records, on the other hand, face the issue of having a low number of cases. For instance, a study across four Nordic countries [27], covering over 23 million residents, included less than 300 events in total. As data could not leave local servers in this study, the model for each country could only be based on an even lower number of events.

Identification of more precise risk factor profiles thus requires larger databases containing all information on relevant covariables, both for individuals developing myocarditis and pericarditis as for those who do not. In some cases, a representative country-wide database may be sufficient to achieve this goal, but only if the population covered in that database is large enough. The latter does not hold for all country-wide databases, as was seen for the Nordic countries [27]. Combining data sources could solve the issue, but this is generally not possible due to privacy concerns and legislation.

Methods that allow for combining data sources without violating privacy concerns or legislations exist, and could be of interest when investigating risk factors for the development of myocarditis and pericarditis following vaccination. For instance, a federated or distributed learning methodology could be applied to develop a statistical model that uses data from several sources without data sharing between these sources [30,31]. Another option would be to estimate coefficients separately for each data source, and to then apply meta-analytical techniques that account for dependencies within and between studies to acquire representative pooled estimates (e.g. [32]). The use of such methods requires interoperable data, which can be achieved through the use of common data models.

While mRNA-type vaccines provide the highest vaccine effectiveness against COVID-19 and are generally safe, they are also associated with the highest risk of myocarditis. A more individual-tailored vaccination approach can be considered where mRNA and ChAdOx1 vaccines are replaced by other vaccine types in those at a certain increased risk of myocarditis and pericarditis. Yet, there is still much to be investigated regarding specific risk factors for the development of myocarditis and pericarditis after COVID-19 vaccination. The identification of more specific risk factors for the development of these diseases is, therefore, of utmost relevance. To achieve this, several practical and methodological challenges, which are mainly due to the low incidence of the outcomes, need to be overcome. It is to researchers to explore methodologies that will help identify which individuals are at highest risk of developing myocarditis and pericarditis, and to data holders to provide data access while complying with the existing boundaries of legislation.
