# Correction: COVID-19 Vaccine-Induced Myocarditis and Pericarditis: Towards Identification of Risk Factors

**DOI:** 10.5334/gh.1276

**Published:** 2024-01-08

**Authors:** Laura C. Zwiers, David S. Y. Ong, Diederick E. Grobbee

**Affiliations:** 1 Julius Clinical, Zeist, The Netherlands; 2 Julius Global Health, Julius Center for Health Sciences and Primary Care, University Medical Center Utrecht, Utrecht University, The Netherlands; 3 Department of Medical Microbiology and Infection Control, Franciscus Gasthuis & Vlietland, The Netherlands

**Keywords:** COVID-19, Vaccination, Myocarditis, Pericarditis

## Abstract

This article details a correction to: Zwiers LC, Ong DSY, Grobbee DE. COVID-19 Vaccine-Induced Myocarditis and Pericarditis: Towards Identification of Risk Factors. Global Heart. 2023; 18(1): 39. DOI: https://doi.org/10.5334/gh.1252

## Correction

This correction relates to the competing interest statement. The original article^1^ stated that the authors have no competing interests to declare. However, it ought to have declared the following:

‘The authors work (part-time) for Julius Clinical Ltd., which is an Academic Contract Research Organization that receives financial support from Moderna Ltd. to conduct research on the cardiovascular safety of COVID-19 vaccination. Moderna had no role in the current paper. Additionally, the third author, D.E. Grobbee, serves as the editor-in-chief of *Global Heart*. However, he was removed from all editorial processes relating to this paper while it was under review.’

## Footnotes

https://globalheartjournal.com/articles/10.5334/gh.1252.
